# Characterization of locomotor activity circadian rhythms in athymic nude mice

**DOI:** 10.1186/1740-3391-11-2

**Published:** 2013-02-01

**Authors:** Natalia Paladino, José M Duhart, Malena L Mul Fedele, Diego A Golombek

**Affiliations:** 1 Laboratorio de Cronobiología, Universidad Nacional de Quilmes, Nacional de Quilmes, R. S. Peña 180, Bernal, Buenos Aires, 1876, Argentina

## Abstract

**Background:**

The relation between circadian dysregulation and cancer incidence and progression has become a topic of major interest over the last decade. Also, circadian timing has gained attention regarding the use of chronopharmacology-based therapeutics. Given its lack of functional T lymphocytes, due to a failure in thymus development, mice carrying the Foxn1^(Δ/Δ)^ mutation (nude mice) have been traditionally used in studies including implantation of xenogeneic tumors. Since the immune system is able to modulate the circadian clock, we investigated if there were alterations in the circadian system of the athymic mutant mice.

**Methods:**

General activity circadian rhythms in 2–4 month-old Foxn1^(Δ/Δ)^ mice (from Swiss Webster background) and their corresponding wild type (WT) controls was recorded. The response of the circadian system to different manipulations (constant darkness, light pulses and shifts in the light–dark schedule) was analyzed.

**Results:**

Free-running periods of athymic mice and their wild type counterpart were 23.86 ± 0.03 and 23.88 ± 0.05 hours, respectively. Both strains showed similar phase delays in response to 10 or 120 minutes light pulses applied in the early subjective night and did not differ in the number of c-Fos-expressing cells in the suprachiasmatic nuclei, after a light pulse at circadian time (CT) 15. Similarly, the two groups showed no significant difference in the time needed for resynchronization after 6-hour delays or advances in the light–dark schedule. The proportion of diurnal activity, phase-angle with the zeitgeber, subjective night duration and other activity patterns were similar between the groups.

**Conclusions:**

Since athymic Foxn1^(Δ/Δ)^ mice presented no differences with the WT controls in the response of the circadian system to the experimental manipulations performed in this work, we conclude that they represent a good model in studies that combine xenograft implants with either alteration of the circadian schedules or chronopharmacological approaches to therapeutics.

## Introduction

Daily environmental changes have imposed a selective pressure for life on Earth, driving the development of a circadian clock mechanism for the generation and entrainment of rhythms in physiological and behavioural variables (e.g. body temperature, hormonal secretion, sleep, locomotor activity, etc.). In mammals, the master clock resides in the hypothalamic suprachiasmatic nuclei (SCN), and the principal signal that adjusts its activity is the light–dark cycle [1,2].

A vast amount of bibliography accounts for the great number of health challenges observed in people working under circadian disruptive routines like the schedules of night- and rotating shift- working or airline workers who perform frequent transmeridian flights in which daily cues are continuously changing. Health problems associated to work-related chronodisruption include cardiovascular and gastrointestinal diseases, metabolic alterations, sleep disorders, and more importantly, elevated cancer rates [3,4]. Indeed, the epidemiological evidence [3,5-9] has led the WHO’s International Agency for Research on Cancer (IARC) to the declaration of shift-working as a relevant risk factor for cancer [10]. Moreover, circadian timing might also be important for cancer therapeutics, since there is growing evidence suggesting that chronopharmacology-based chemotherapies approaches may benefit the outcome of cancer treatments and reduce drug toxicity (reviewed in [11]).

Although there is substantial information regarding the circadian modulation of many immunological variables [12-14], little is known about the possible effect of immune factors on the circadian system itself. Several reports suggest a possible immune feedback regulation of the circadian clock. For instance, the sleep–wake cycle (one of the most evident circadian rhythms) is modified by proinflammatory cytokines [15] and systemic low doses of LPS administered at circadian time (CT) 15 (CT12 corresponds to locomotor activity onset) produce a photic-like phase delay of locomotor rhythm in mice, that depend on Toll-like receptor-4 signaling [16,17].

Foxn1^(Δ/Δ)^ mutant –nude- mice have a specific defect in thymic development, characterized by a block in thymic epithelial cells differentiation at an intermediate progenitor stage, resulting in the production of abnormally functioning T cells [18]. The nude mice are frequently used in cancer research because the feasibility to implant xenogeneic tumor cells (and, in particular, human tumor cells lines [19]). Given the increasing interest in the effects of circadian disruption on cancer biology [20-25] and the extended use of athymic mice as an useful tool for cancer studies, we aimed to characterize the circadian rhythms in nude and their corresponding control strain mice (Swiss Webster). Although the effects of the athymic mutation on some characteristics of circadian biology have been previously reported [26], our work extend these findings, analyzing circadian rhythmicity in constant conditions, and the response to shifts in the light/dark cycle and to light pulses. This approach results relevant to control the circadian variables in cancer-related experiments, and, in addition, these experiments assess the impact of a specific immune defect on the mammalian circadian system.

## Materials and methods

### Animals

Adult (2 months old) Foxn1^(Δ/Δ)^ mutant and wild type (WT) Swiss Webster (N:NIHS) male mice (Mus musculus) were housed in single cages under a 12:12-h light:dark photoperiod (LD, with lights on at 8 AM and light off at 8 PM) or in constant dark (DD) conditions with food and water access ad libitum. For the experiments in constant darkness, mice were transferred to DD conditions 20 days prior to the treatments. Foxn1^(Δ/Δ)^ animals received sterile air ventilation, as well as sterile food and water. All animal experiments were carried out in accordance with the NIH Guide for the care and use of laboratory animals.

### Behavioral analysis

Mice were housed in individual cages equipped with infrared sensors, their locomotor activity circadian rhythm was recorded with a system designed in our laboratory and counts were collected every 5 min. Time is expressed as zeitgeber time (ZT), with ZT12 defined as the time of lights off in LD conditions, or circadian time (CT), with CT12 defined as the onset of locomotor activity in DD. Manipulation in DD conditions were performed under dim red light (<1 lux).

For light pulses experiments, mice maintained in DD were exposed to a 10 min- or 2 hour-white light pulse of 150 lux at CT15 or CT22. The onset of activity at CT12 was used as a phase reference point to calculate phase shifts.

Abrupt 6-h advances in the LD schedule were achieved by advancing the time of lights-on and shortening of the dark phase. Conversely, abrupt 6-h delays in the LD schedule were achieved by delaying the time of lights-on, and lengthening the dark phase.

### Immunohistochemistry

Mice were deeply anaesthetized with a cocktail containing ketamine (150 mg/kg) and xylazine (10 mg/kg) 60 min after light pulse at CT15 and perfused intracardially with 4% paraformaldehyde in 0.01 M phosphate buffer. Brains were carefully removed, post-fixed overnight, cryoprotected in sucrose 30% in 0.01 M PBS for 24 h and 25 μm thick coronal sections were cut with a freezing microtome and collected in 0.01 M PBS. Free floating slices were blocked with 5% non-fatty milk in PBS contained 0.4% Triton X-100 and incubated with primary antisera raised in rabbit against c-Fos (Santa Cruz Biotechnology, 1:2000) diluted in the same solution, for 48 hs at 4°C. Sections were then treated using the avidin–biotin method with a Vectastain Elite Universal kit containing a biotinylated universal secondary antibody, avidin and biotilylated horseradish peroxidase (Vector Laboratories, Burlingame, CA) and Vector-VIP peroxidase substrate (SK-4600). Image analysis and cell counting was performed using ImageJ software, as described previously [27].

### Data analysis

All circadian parameters were calculated using El Temps program (Antoni Díez Noguera, University of Barcelona). For phase angle (ψ) calculation and phase shifts analysis, individual waveforms were performed using 15 consecutive days in LD or DD condition. Activity onsets were defined as the moment in which the activity curve is on top of the mean line of each individual waveform. ψ was calculated as the difference between the activity onset in LD conditions and the time of lights off.

In order to improve the visualization and analysis of the activity patterns we computed an hourly activity profile, in which we plotted mean locomotion for each hour (or circadian hour) relative to average activity counts. These individual hourly waveforms were used for the analysis of activity distribution both in LD and DD conditions. Also, an average waveform for each animal group was calculated using these individual waveforms.

The activity distribution over the day in LD conditions was analyzed in four different intervals: ZT12-23, ZT23-3, ZT3-10 and ZT10-12 (see results). The contribution of each interval to total activity was calculated by performing hourly waveform analysis of the relative activity for each individual animal, and measuring the area under the curve for each interval. The ratio between this area and the total area under the curve of the waveform, expressed as a percentage, was used for further comparisons.

Resynchronization to a new LD cycle, after 6-hour phase shifts of the time of lights off, was considered fully accomplished when activity onset took place at the new time of lights off ±15 minutes. We calculated the number of days (transients) required to resynchronize for each animal.

In DD conditions, free-running activity periods (τ) were determined by Chi-square periodograms. Duration of the subjective night (α), was calculated by performing an hourly waveform analysis of the relative activity data for each animal in DD (using the corresponding τ), and measuring the time length covered by the portion of the curve on top of the mean of activity. Conversely, ρ (duration of the subjective day) was calculated as the time length covered by the portion of the curve under the mean of activity. The percentage of total activity occurring during α was calculated as the percentage of the area under the curve of the waveform analysis occurring within the α interval.

Phase shifts were calculated as follows: individual waveforms of the ten previous days to the light pulse and of ten days after the light pulse (excluding the 2 cycles immediately after the pulse) were obtained; the time at which the activity curve crossed the mean line of each waveform was defined as the activity onset and used as phase marker of the rhythm prior and after the pulse. Phase shifts were calculated as the difference between the two onsets.

Data are presented as mean ± SEM. Differences between the two groups were analyzed by unpaired Student’s *t* test or the non-parametric Mann Whitney test, accordingly. P values of 0.05 or less were considered to be statistically significant.

## Results

### Free running period and general activity pattern in nude mice

We first analyzed the characteristics of the circadian activity pattern in LD and DD conditions of nude mice and their respective control WT strain (Swiss Webster N:NIHS). We evaluated the ψ between the time of lights off and the beginning of the locomotor activity. The activity onset occurred after the time of lights off in both strains of mice, without differences between the ψ in nude and control mice (8.7 ± 6.3 min, n = 9 and 8.13 ± 3.14, n = 6, respectively; p > 0.05, Student’s *t* test; Table 1).

**Table 1:** Activity patterns in LD conditions

		**Nude**	**WT**	**P**
		**(mean ± SEM)**	**(mean ± SEM)**	
**ψ (min)**		8.7 ± 6.3	8.1 ± 3.1	ns
	Interval			
**%Area**	ZT12-23	64.54 ± 3.43	65.68 ± 6.82	ns
	ZT23-3	12.37 ± 2.17	14.81 ± 4.00	ns
	ZT3-10	13.49 ± 1.69	10.22 ± 1.75	ns
	ZT10-12	8.11 ± 1.13	6.85 ± 1.71	ns

Phase angle (ψ, difference of activity onset with the time of lights off) and percentage of activity for each ZT interval over total activity were calculated (% Area, see methods for details). P values correspond to Student’s *t* test between nude (n = 9) and WT (n = 6) control mice. Data are expressed as mean ± SEM.

**Figure 1:**
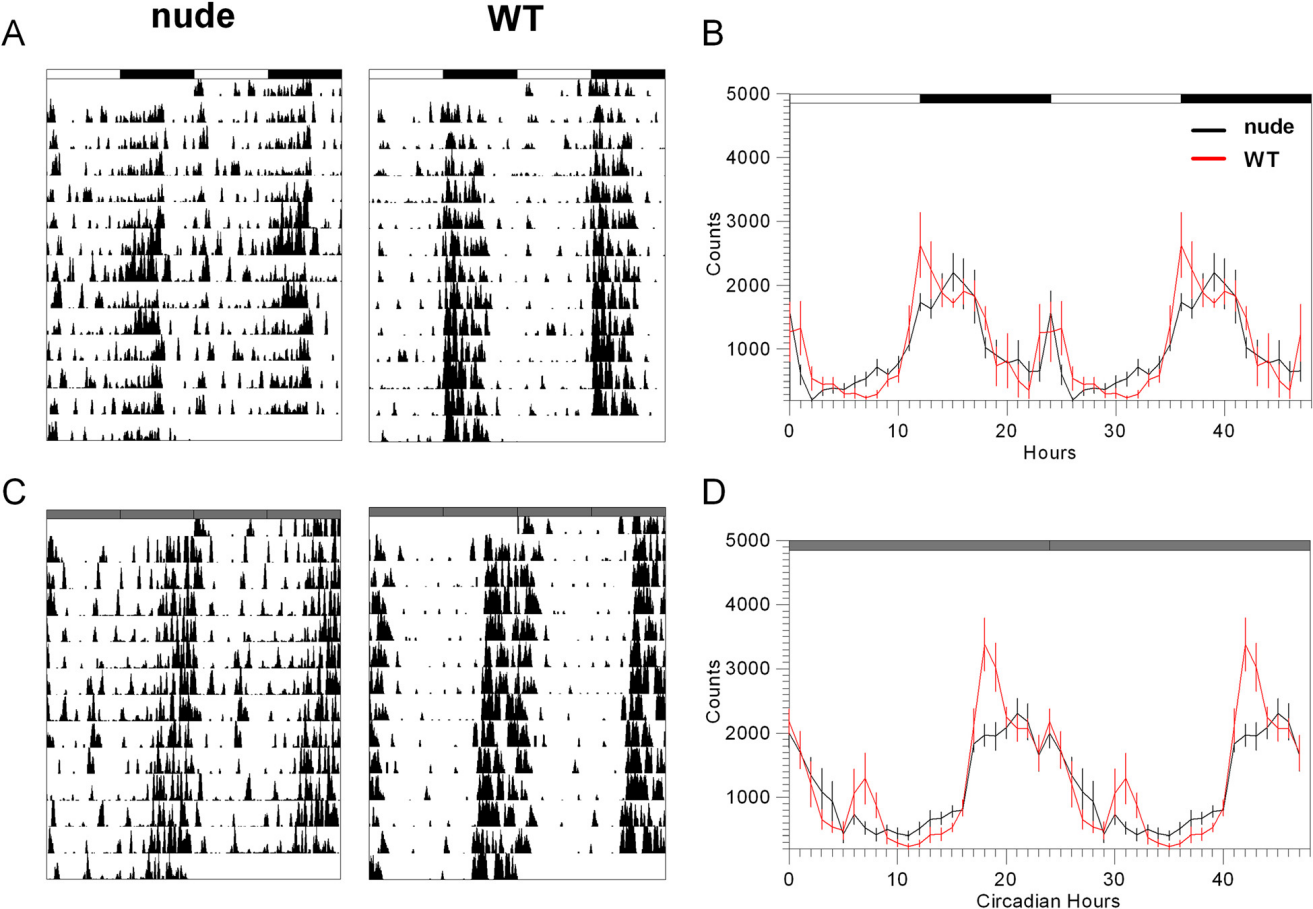
**Activity patterns of nude and WT mice in LD and DD conditions.** Representative actograms of nude (left) and wild-type (right) mice under LD (**A**) and DD (**C**) conditions. Average waveforms of nude (black) and WT (red) mice for LD (**B**) and DD (**D**) condition were calculated using the individual waveforms of relative activity.

The general pattern of activity in LD condition was similar in both strains, exhibited nocturnal activity and a bout of activity occurring after the time of lights on, which lasted for about 2–3 h (Figure 1A, B). Also, about 50% of the WT mice showed a bout of anticipatory activity, occurring 1–2 hours previous to lights off (data not shown). In order to analyze the contribution of each activity bout to the general activity profile of both mouse groups, we calculated the percentage of total activity corresponding to ZT12-23, ZT23-3, ZT3-10 and ZT10-12. We found no significant difference in the percentage of total activity corresponding to each of the intervals previously measured, with strictly nocturnal activity (ZT12-23) accounting for approximately 68% of the total activity in both strains (p > 0.05, Student’s *t* test; Table 1).

The general pattern of activity in DD condition was also similar in both mice strains. However, the activity bout that occurred after the time of lights on in LD conditions, tended to disappear under constant darkness in some mice, more often in the nude strain (Figure 1C, D). We next evaluated the period of locomotor activity in DD condition. There were no statistically significant differences between the two strains (p > 0.05, Student’s *t* test; Table 2). The period was 23.86 ± 0.03 circadian hours -ch (1431 ± 1.9 min, n = 9) in nude and 23.88 ± 0.05 ch (1436 ± 3.1 min, n = 6) in WT mice. We found no statistically significant difference in α (8.7 ± 0.6 hours, corresponding to the 36% of the circadian period for nude mice, n = 9; and 10.8 ± 1.5 hours, corresponding to the 45% of the circadian period for control mice, n = 6; p > 0.05, Student’s *t* test; Table 2). However, we found that nude mice had a smaller percentage of total activity occurring during the α interval than the WT control (66.1 ± 3.5% for nude mice and 79.9 ± 3.7% for WT controls, p < 0.05, Student’s *t* test, n = 9 for nude mice and n = 6 for WT mice, Table 2). Also, there were no differences in ρ between the two strains, with duration of 15.3 ± 0.6 and 13.0 ± 1.4 hours for nude and WT mice, respectively and α/ρ ratio did not statistically differ between the two groups (0.58 ± 0.06 and 0.98 ± 0.27 for nude and WT mice, respectively, Table 2).

**Table 2:** Activity patterns in DD conditions

	**Nude**	**WT**	**p**
**τ (hours)**	23.86 ± 0.03	23.88 ± 0.05	ns
**α (hours)**	8.7 ± 0.6	10.8 ± 1.5	ns
**% α/τ**	36.5 ± 2.4	45.4 ± 6.3	ns
**% Area (α)**	66.1 ± 3.5	79.9 ± 3.7	< 0.05
**α/ρ**	0.58 ± 0.06	0.98 ± 0.27	ns

Free running period (τ), α (time duration of the subjective day) and ρ (time duration of the subjective night) were calculated as described in methods. τ (hours), total α length (hours), α/τ ratio expressed as percentage, (% α/τ), percentage of total activity occurring during α (% Area (α)) and the α/ρ ratio are expressed as mean ± SEM for nude (n = 9) and WT (n = 6) mice. P values correspond to Student’s *t* test between the two mice groups.

### Characterization of circadian responses to light in nude mice

**Figure 2:**
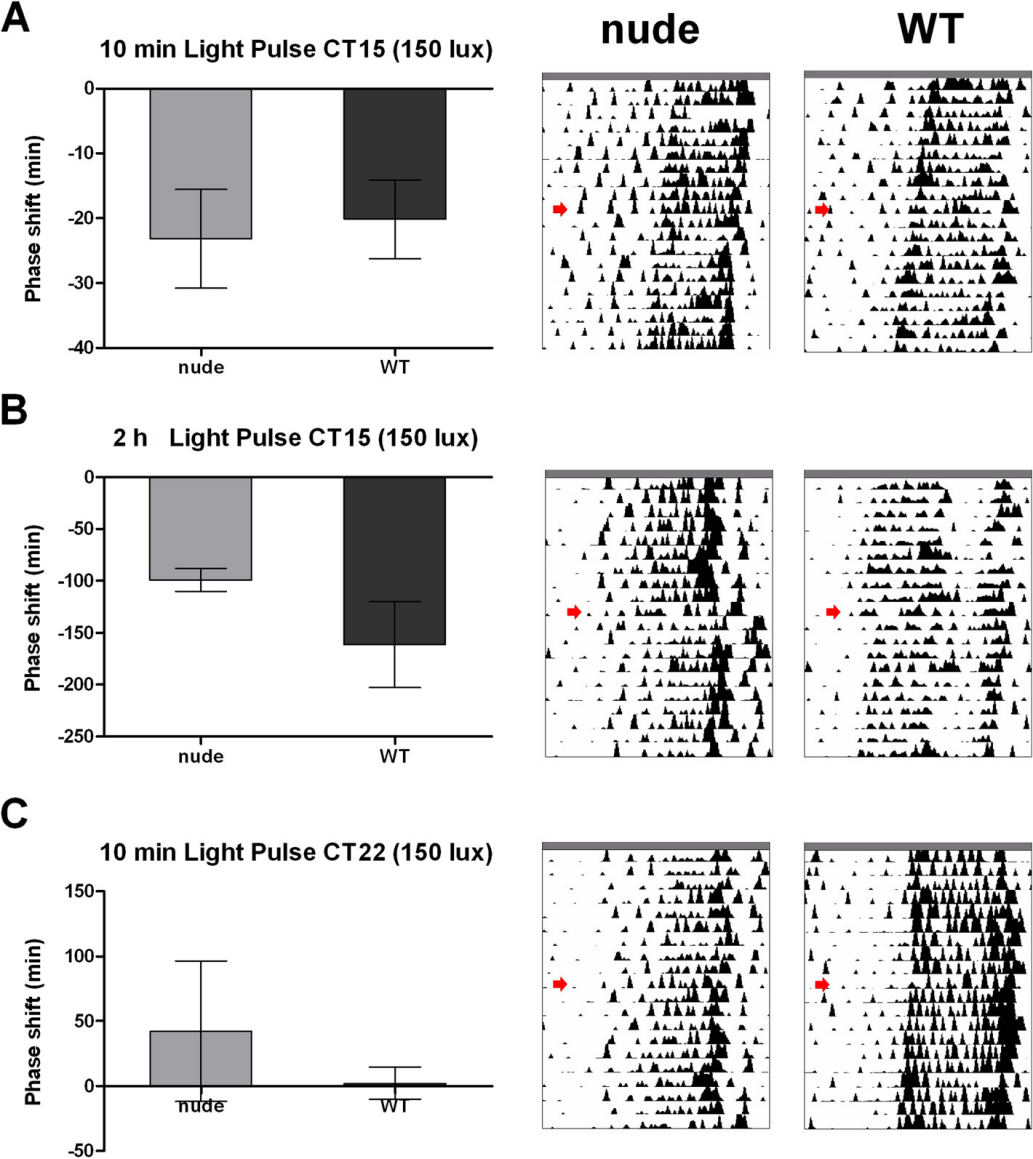
**Response to light pulses in nude and WT mice.** Athymic nude and control WT mice were subjected to light pulses during the early night (**A**, **B**) or the late night (**C**). **A.** Representative actograms of general activity of nude and WT mice receiving a light pulse (150 lux, 10 min) at CT15 (Right). No significant difference was found between the two strains (Left, mean ± SEM, -23.2 ± 6.2 min in nude and −20.2 ± 6.0 min in WT mice, n = 6, p > 0.05, Student’s *t* test). **B.** Representative actograms of nude and WT mice receiving a light pulse (150 lux, 120 min) at CT14 (Right). No significant difference was found between the two strains (Left, mean ± SEM, -99 ± 6.3 min in nude and −161.3 ± 29.3 min in WT mice, n = 3, p > 0.05, Mann Whitney test). **C.** Representative actograms of nude and WT mice receiving a light pulse (150 lux, 10 min) at CT22 (Right). No significant difference was found between the two strains (Left, mean ± SEM, 42 ± 31 minutes for nude and 2 ± 12 for WT mice, n = 3, p > 0.05, Mann Whitney test).

We next analyzed the circadian responses to different light treatments in both nude and WT mice. Under DD conditions, light pulses delivered in the early subjective night (CT15) induce phase delays in behavioral rhythms controlled by the clock, while late night pulses (CT22) advance the circadian phase. To evaluate this circadian response to light, mice were exposed to 10 min-150 lux white light pulse at CT15 and CT22 and the corresponding phase-shift were calculated. At CT15 we observed a phase delay of −23.2 ± 6.2 min (n = 6) in nude mice and −20.2 ± 6.0 min (n = 6) in controls, with no statistically significant difference between the two groups (p > 0.05, Student’s *t* test; Figure 2A). Since light pulses of the same intensity and duration at early night usually produce larger phase delays (40–150 min) in other mice strains [16,28], we analyzed the effects of a 2 h light pulse applied between CT14 and CT16. Such pulses induced a phase delay of −99 ± 6.3 minutes in nude and of −161.3 ± 29.3 minutes in WT mice, that did not differ statistically between groups (p > 0.05, Mann Whitney test, n = 3; Figure 2B). Light pulses administered at CT22 produced no significant difference between the phase shifts presented by both strain (42 ± 31 min for nude and 2 ± 12 min for WT mice; p > 0.05, Mann Whitney test, n = 3; Figure 2C). Moreover, light pulses did not change the overall activity distribution throughout the circadian day.

**Figure 3:**
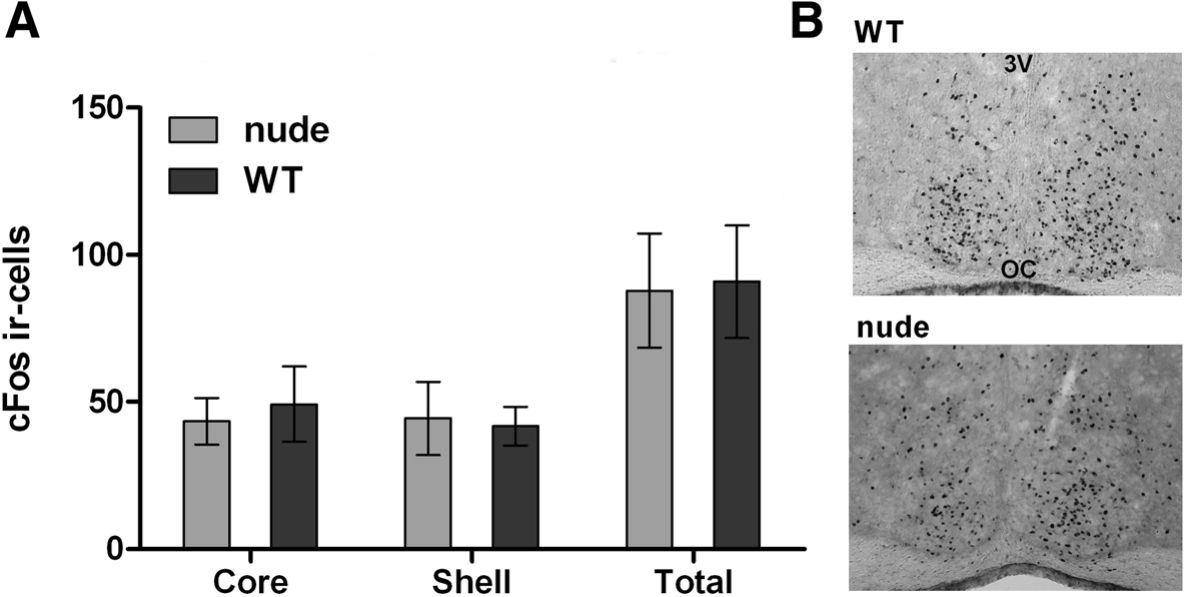
**Light-induced cFos expression in the SCN of nude and WT mice. A.** Mean ± SEM of positive cells number in the ventrolateral (core), dorsomedial (shell) and total areas of the SCN of nude and WT mice 60 min after a 10 min light pulse at CT15 (no significant difference was found between the number of cells either in the core, shell or total areas of the SCN; n = 3, p > 0.05, Mann Whitney test). **B.** Representative SCN coronal sections illustrating c-Fos expression in nude and WT mice exposed to 10 min light pulse (150 lux) at CT15.

We also analyzed the expression of c-Fos in hypothalamic coronal brain sections 60 min after a 10 min light pulse at CT15. There was no difference between the number of cells expressing c-Fos in WT and nude mice (p > 0.05, Mann Whitney test, n = 3; Figure 3).

**Figure 4:**
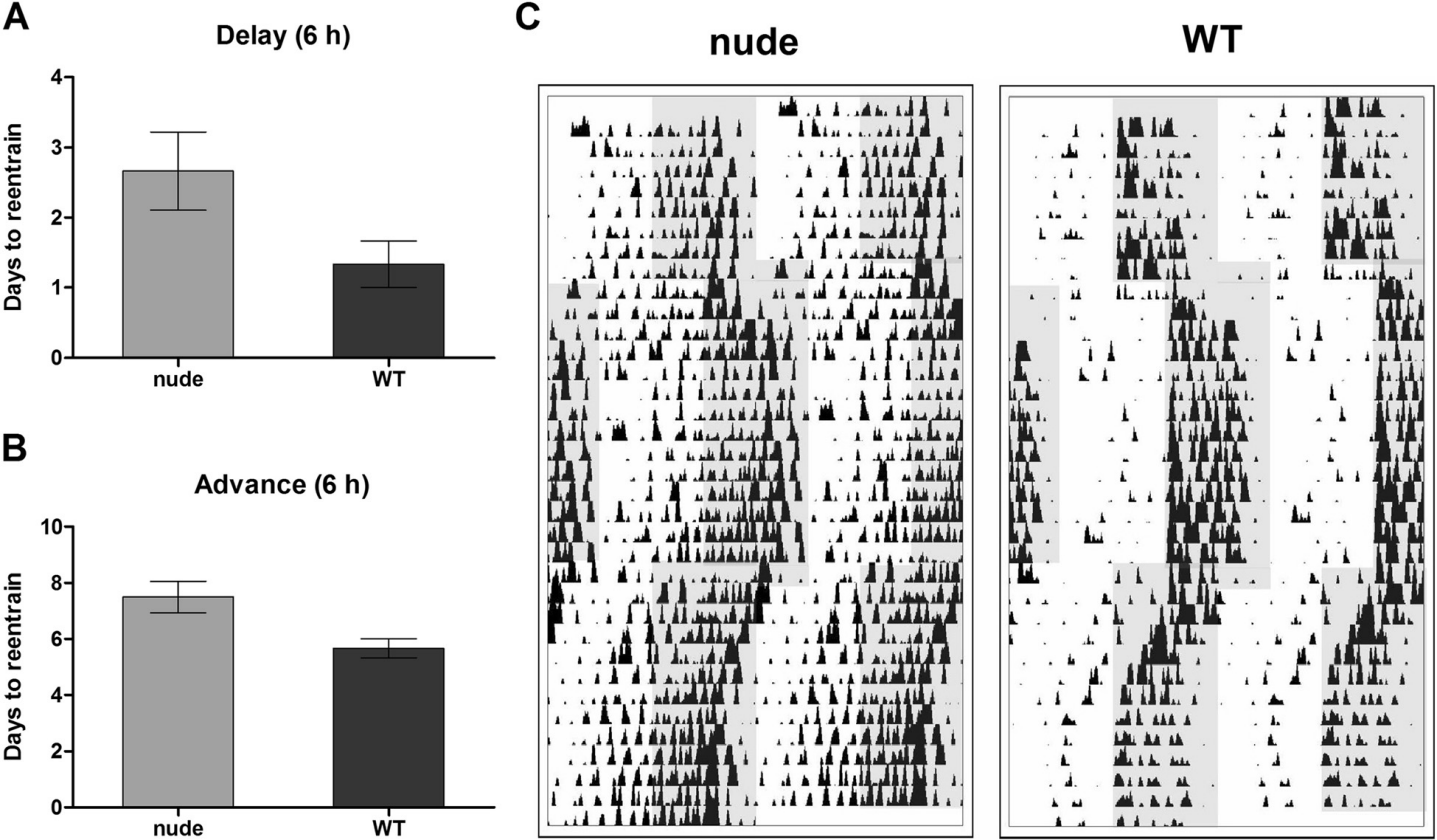
**Resynchronization to phase shifts in the light–dark schedule.** Nude and WT control mice were subjected to a 6-h shift in the light–dark schedule, and the transient needed to resynchronize to the new schedule was quantified. **A.** Time (days) needed to reentrain to the light–dark cycle in nude and WT mice subjected to a 6-h phase delay (2.7 ± 0.5 and 1.3 ± 0.2 days for nude and WT, respectively; p > 0.05 Mann Whitney test). **B.** Transient (days) needed to reentrain after 6-h phase advance of the LD cycle (7.5 ± 0.5 and 5.7 ± 0.2 days for nude and WT, respectively; p > 0.05 Mann Whitney test). **C.** Representative actograms of nude and WT mice subjected first to a 6-h phase delay and then (15 days later) to a 6-h phase advance of the LD cycle. Grey background represents time of lights-off.

To evaluate the resynchronization capability of the mice groups, we delayed or advanced the time of the LD cycle by 6-h and calculated the transient needed to entrain to the new cycle. Nude mice exhibited a non-significant tendency towards larger transients for reentrainment. For *delay* phase shifts nude and WT mice transients were of 2.7 ± 0.5 (n = 6) and 1.3 ± 0.2 (n = 3) days, respectively. In the *advances* phase shift, nude mice took 7.5 ± 0.5 (n = 6) while WT mice took 5.7 ± 0.2 (n = 3) days to reentrain to the new schedule (p > 0.05, for both, delays and advances, Mann Whitney test; Figure 4).

## Discussion

Studies using xenogeneic tumor implantation in athymic immune-deficient mice are becoming a useful tool for understanding both the importance of circadian dysregulation as a factor that potentiates tumor progression and the benefits of timed cancer therapies [29-31]. Therefore, a circadian characterization of nude mice results necessary to fully comprehend the results of such experimental approaches. A previous report by Beau et al. (1990) has described the patterns of circadian locomotor activity under LD conditions, analyzing 3 days of recording. In the present study, we extend those results, analyzing circadian rhythms in constant conditions, the response to light pulses and the resynchronization to light–dark cycles shifts.

Our results show that general activity characteristics (ψ and activity distribution over different ZT intervals) in athymic mice were not different from their WT control. Presence of anticipatory behavior was seen in approximately 50% of the WT mice, evidenced by a sharp bout of activity occurring 1–2 hours before the time of lights off (ZT10-12). However, the percentage of total activity occurring within this interval did not differ between athymic and control mice, probably due to the presence of irregular diurnal activity also during this interval in the nude mice. An important activity bout was found in both WT and mutant mice, occurring in an interval ranging from ZT23 to ZT3. In a previous report showing an activity waveform for WT Swiss Webster mice, this activity bout was also found in the first hours of the light phase, and was accompanied by a raise in body temperature, suggesting that it might be a characteristic of the Swiss background [32]. A reduced amount of global activity has been described in nude mice from C57BL/6J background, compared with their WT controls [26]. Unfortunately, due to differences in the sensitivity among infrared sensors, the absolute number of counts is not reliably comparable between different animals, therefore making it impossible to estimate a global activity mean that can be used to compare the nude and the WT strains.

Under conditions of continuous darkness (DD), circadian rhythms assume free-running periods that are close to 24 hours. Our data indicate a slightly larger period in nude and control mice with Swiss Webster background (23.86 and 23.88 h, respectively), compared with a previous report that showed a 23.3 h period in wheel running activity rhythms for the Swiss strain mice [33]. However, it has been described that wheel-running can shorten the period of circadian rhythms in rodents [34-36], which might explain the longer period found in our work, where we measured general activity rhythms of mice without access to running wheels. Importantly, there were no differences between the free-running period or in the α in DD of nude and WT control mice (although the percentage of activity occurring within the α interval was lower in nude mice), suggesting that the Foxn1^(Δ/Δ)^ mutation doesn’t affect the central pacemaker, at least regarding this particular behavioral output. In addition, our findings add substantial information regarding the circadian system of Swiss mice, a strain that has received relatively little attention in chronobiology.

Circadian entrainment to light–dark cycles is mediated through daily phase shifts induced by light on the pacemaker. We found no differences in the magnitude of the phase shifts (both at the early or late night) between WT Swiss mice and the nude mutant mice. Also, the ability to resynchronize to a 6-hour shift (both in delayed or advanced schedules) in the LD cycle did not differ between the two strains. This finding could be expected given the similarities in both the response to light pulses and the endogenous periods of each strain.

Alterations in the immune system can produce changes in circadian physiology. For example, mice mutant for the Galectin-1 gene, which regulates cytokine synthesis [37], show altered free-running periods and responses to early-night light pulses [38]. Moreover, activation of the immune system can also modulate the circadian clock. Peripheral LPS administration or introcerebroventricular (icv) injection of TNF-α or IL-1β produced phase delays in wheel running activity [17,39], and icv delivery of a proinflammatory cocktail also activated c-Fos expression in the SCN [40]. In addition, clock gene expression can be altered by exposure to different proinflammatory factors [41-44]. Besides the lack of functional T cells, nude athymic mice show resistance to *Bacillus anthracis*, *Listeria monocytogenes* and *Staphylococcus aureus* infection [45,46], reduced levels of circulating TNF-α, IL-6 and IFN-γ, in response to *Pseudomonas aeruginosa* exotoxin [47] and present a higher activation of macrophages [48]. It should also be noted that the nude mice were found to have a reduced number of glial cells (oligodendrocytes, astrocytes, and microglia), with presence of hypertrophic astroglia in the adult brain [49], and glial cells have been suggested to modulate the circadian system [50-52]. However, given the results of this work, none of these characteristics (or other phenotypic changes) occurring in the Foxn1^(Δ/Δ)^ mice results in a notable alteration in the circadian rhythm of activity.

## Conclusions

The results of the present work show that the profound alterations in the immune system of the nude mice do not modify the circadian activity rhythms of these animals, suggesting that the lack of functional T-cell generation (or any other differential phenotype occurring in the athymic mice) does not produce changes in the circadian control of locomotor activity. Indeed, the effect of the Foxn1^(Δ/Δ)^ (nude) mutation on other circadian rhythms remains to be established. These findings are relevant, both regarding the growing interest in the modulation of the circadian system by immune factors, as well as in the use of the immunodeficient nude mice in cancer research, particularly in approaches that rely in the alteration of normal circadian rhythmicity or in chronopharmacological treatment designs.

## Competing interests

The authors declare that they have no competing interests.

## Authors’ contributions

NP & JMD performed the behavioral experiments and analysis. NP & MMF performed the immunohistochemistry. NP, JMD & DAG conceived the study, participated in its design and drafted the manuscript. All authors read and approved the final manuscript.
